# Multilocus Inherited Neoplasia Alleles Syndrome in a Patient With BRCA2‐Associated Breast Cancer and MLH1‐Related Lynch Syndrome

**DOI:** 10.1155/crom/3353129

**Published:** 2026-04-19

**Authors:** Vaneza Avila-Rodriguez, Alejandro Ruiz-Patiño, Carlos Bonilla-Gonzalez, María Eugenia Manrique Acevedo, Patricia Lopez, Magda Jimena Vargas Diaz, Diego Rubio, María Alejandra Bravo Garzon, Sandra Franco, William Mantilla

**Affiliations:** ^1^ Inpatient Unit, Luis Carlos Sarmiento Angulo Cancer Treatment and Research Center (CTIC), Bogotá, Colombia; ^2^ GIGA Research Group, CTIC/El Bosque University, Bogotá, Colombia; ^3^ Foundation for Clinical and Applied Cancer Research—FICMAC, Bogotá, Colombia; ^4^ Gastrointestinal Cancer Unit, Luis Carlos Sarmiento Angulo Cancer Treatment and Research Center (CTIC), Bogotá, Colombia; ^5^ Pathology Unit, Keralty Group, Bogotá, Colombia; ^6^ Pathology Unit, Luis Carlos Sarmiento Angulo Cancer Treatment and Research Center (CTIC), Bogotá, Colombia; ^7^ Breast Cancer Unit, Luis Carlos Sarmiento Angulo Cancer Treatment and Research Center (CTIC), Bogotá, Colombia

**Keywords:** BRCA2 germline variant, breast cancer, Lynch syndrome, MINAS

## Abstract

Multilocus inherited neoplasia alleles syndrome (MINAS) is a rare but increasingly recognized entity characterized by germline pathogenic variants in multiple cancer susceptibility genes, leading to overlapping hereditary cancer syndromes. The growing use of next‐generation sequencing (NGS) and comprehensive genetic testing has increased MINAS detection, with an estimated 1.37% prevalence among hereditary cancer patients. Genes commonly implicated include BRCA1, BRCA2, MLH1, MSH2, MSH6, PMS2, APC, TP53, PTEN, and STK11, conferring a higher risk of multiple primary malignancies. We describe a case of a postmenopausal woman initially diagnosed with Stage IIIB luminal A breast carcinoma, who developed contralateral breast recurrence with supraclavicular and pulmonary metastases, responding completely to ribociclib and letrozole. Given her early‐onset breast cancer, genetic testing of her hereditary cancer risk revealed BRCA2 and MLH1 germline variants, confirming MINAS syndrome. Subsequent evaluation identified colonic adenocarcinoma (Stage IIIB, MLH1/PMS2 deficiency), leading to total colectomy, hysterectomy, and bilateral salpingo‐oophorectomy. Surveillance was proposed for colon cancer, while ribociclib and letrozole were continued for breast cancer. This case highlights the clinical complexity of MINAS syndrome. The comprehensive genomic profiling is critical for guiding targeted therapy, immunotherapy, and surgical decision‐making, optimizing outcomes in this high‐risk population.

## 1. Introduction

The coexistence of multiple hereditary cancer syndromes is acknowledged as multilocus inherited neoplasia alleles syndrome (MINAS); it is a rare condition in which pathogenic variants in two or more cancer‐predisposing genes lead to overlapping syndromic hereditary cancer features within a single individual with a higher risk of developing multiple primary malignancies [1]. The prevalence of MINAS is relatively low, but it is increasing due to the recognition that genetic testing is becoming more comprehensive [2]. In a study involving a large cohort of patients with hereditary cancer, 1.37% were found to have two pathogenic variants in dominant cancer‐predisposing genes [3]. MINAS may result in overlapping hereditary cancer syndromes that include hereditary breast and ovarian cancer syndrome (BRCA1, BRCA2), Lynch syndrome (MLH1, MSH2, MSH6, PMS2), familial adenomatous polyposis (APC), Li–Fraumeni syndrome (TP53), Cowden syndrome (PTEN), and Peutz–Jeghers syndrome (STK11) [4]. Recent advancements in next‐generation sequencing and comprehensive genetic testing have enhanced the understanding of MINAS, providing critical insights into its prevalence, molecular mechanisms, and clinical implications [5]. While some cases of MINAS may exhibit severe clinical manifestations, particularly when involving DNA damage repair‐associated genes, the overall evidence does not consistently support more severe phenotypes at diagnosis. These findings support considering MINAS in patients with atypical inherited cancer syndrome phenotypes, particularly when the personal or family history cannot be fully explained by a single hereditary cancer predisposition syndrome. The identification of MINAS highlights the intricate interplay between tumor biology, inherited genetic variants, and environmental factors, emphasizing the need for integrative and personalized therapeutic strategies [6].

In this report, we present a case of a postmenopausal woman patient, initially diagnosed with Stage IIIB luminal A breast carcinoma, who experienced disease relapse and was later found to have colon adenocarcinoma, with genetic testing of hereditary cancer revealing germline BRCA2 and MLH1 variants consistent with MINAS.

## 2. Case Report

**Figure 1:**
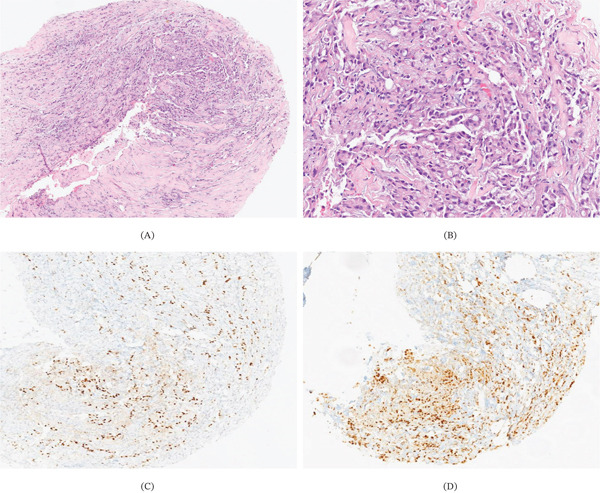
Biopsy of the left supraclavicular mass: (A, B) (Hematoxylin‐eosin): Soft tissues are infiltrated by a moderately differentiated ductal‐type carcinoma. (C) IHC estrogen receptors show strong nuclear staining in 90% of tumor cells. (D) IHC GATA‐3 shows strong nuclear positivity in tumor cells Histopathological findings of the left supraclavicular mass biopsy: (A, B) Soft tissue infiltration by a malignant epithelial neoplasm was observed, composed of atypical epithelial cells with moderate nuclear pleomorphism and prominent nucleoli, forming occasional duct‐like structures. (D) Immunohistochemical studies revealed strong nuclear positivity for GATA‐3 and E‐cadherin in tumor cells. (C) Estrogen receptors demonstrated strong nuclear staining in 90% of tumor cells, while progesterone receptors were strongly positive in 30% of tumor cells. HER2 oncoprotein expression was equivocal (Score 2+), and the Ki‐67 proliferation index was 10%. Based on these findings, the diagnosis was consistent with metastatic involvement by an invasive ductal carcinoma of no special type, nuclear Grade 2, of breast origin. MMR IHC was performed, showing preserved expression of mismatch repair proteins PMS2, MSH2, MSH6, and MLH1.

**Figure 2:**
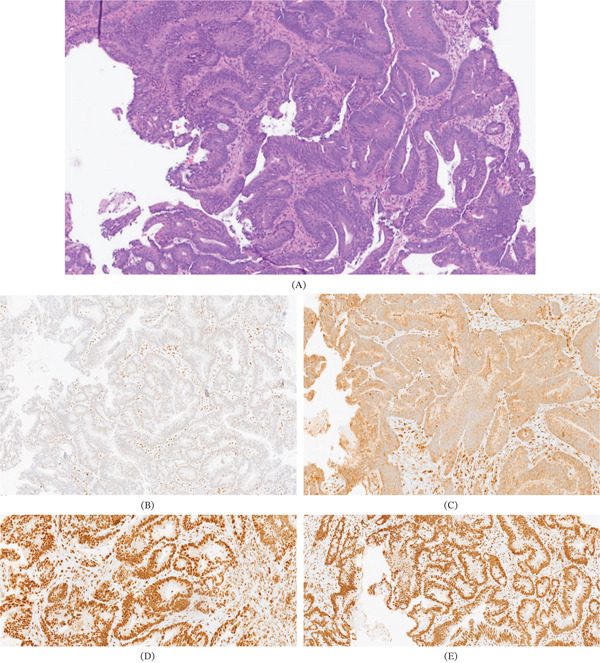
Biopsy of the colon. (A) Moderately differentiated intestinal‐type colonic adenocarcinoma. (B) Loss of nuclear expression of MLH1 protein. (C) Loss of nuclear expression of PMS2 protein. (D, E) Preserved nuclear expression of MSH2 and MSH6 proteins. Histopathological findings of the cecal biopsy: (A) The colonic mucosa infiltrates a malignant epithelial neoplasm composed of irregular glandular structures lined by columnar cells exhibiting nuclear hyperchromasia, prominent nucleoli, and frequent mitotic figures. The glands are arranged in a back‐to‐back pattern, consistent with a moderately differentiated intestinal‐type colonic adenocarcinoma. Immunohistochemical analysis revealed strong positivity for CDX2. HER2 oncoprotein expression was negative (Score 0). (B, C) Microsatellite instability studies demonstrated loss of nuclear expression of MLH1 and PMS2, (D, E) with preserved nuclear expression of MSH2 and MSH6. The loss of MLH1 and PMS2 expression by immunohistochemistry may suggest either a germline variant or somatic promoter hypermethylation of the MLH1 gene.

A 53‐year‐old postmenopausal patient presented with Stage IIIB (cT4bN1M0) luminal A ductal carcinoma of the left breast. After neoadjuvant chemotherapy (AC schema followed by taxane), she underwent mastectomy and axillary lymph node dissection. Surgical pathology reported a Grade 3, poorly differentiated infiltrating carcinoma (NOS) with an apocrine‐like component and involvement of 8/24 nodes (ER/PR+ 70%, HER2 0+, Ki67 40%). There was no complete pathological response; however, the patient declined further adjuvant treatment. After three years, the patient presented a relapse of the disease documented in images of a new right breast lesion (contralateral breast), supraclavicular nodal lymph, and lung nodules. A biopsy was performed in the lung and breast without malignant pathology, and supraclavicular nodal lymph showed metastasis of breast cancer with pathology report of ductal infiltrating carcinoma, NOS, Grade 2, ER (+) 90%, PR (+) 30%, HER2 2+ (FISH: negative), Ki67 10%, and MMR was proficient (Figure 1). The consideration was a relapse of the breast cancer, and the first line of systemic treatment for metastatic disease was with cyclin‐dependent 4/6 kinase inhibitor (CDKi 4/6), ribociclib, and aromatase inhibitor letrozole, and 4 cycles of treatment were completed. Therefore, a complete response was evidenced in images of evaluation. Nevertheless, considering a patient with breast cancer before 50 years old and two primary cancers, genetic testing (MyRisk) was performed and reported BRCA2 c.1378_1382del and MLH1 c.790+1G>A variants, classified as pathogenic/likely pathogenic according to the ACMG/AMP criteria. Therefore, colonoscopy was performed with evidence of neoplasm of the cecum, and the reported pathology was moderately differentiated adenocarcinoma, with loss of expression of MLH1 and PMS2, intact expression of MSH2 and MSH6 by immunochemistry, and the diagnosis of localized colon adenocarcinoma was established (Figure 2). Furthermore, endometrial thickening was documented in the pelvic magnetic resonance imaging.

**Figure 3:**
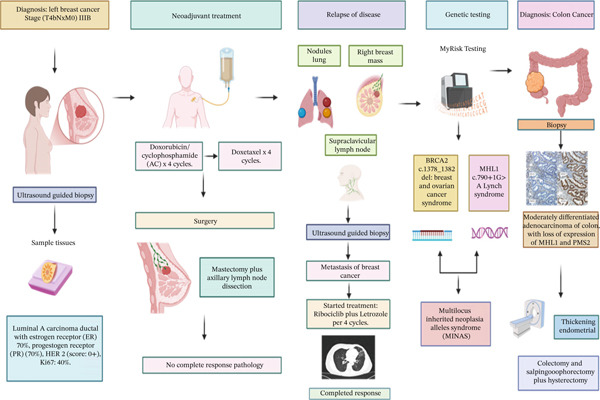
Timeline of evolution, clinical, and response to treatment. Created in https://biorender.com

This patient has two hereditary syndromes: breast and ovarian cancer syndrome associated with the BRCA2 variant, and Lynch syndrome. The coexistence of both hereditary syndromes is considered MINAS. Therefore, this patient with an early disease of colon cancer and a high risk of endometrial cancer due to Lynch syndrome will undergo surgical treatment with total colectomy plus ileorectal anastomosis and salpingo‐oophorectomy plus hysterectomy. The surgical pathology was reported as Grade 2 moderately differentiated colon mucoproducing adenocarcinoma with lymphovascular compromise, perineural invasion, with 0/37 of negative lymph nodes for tumor with pT3N1cM0 corresponding to Stage IIIB. Therefore, ribociclib and letrozole were continued for the treatment of breast cancer, and surveillance was proposed for the treatment of colon cancer with deficient MMR, and in the case of relapse, the option of treatment will be immunotherapy (Figure 3).

## 3. Discussion

This case presents a complex clinical scenario involving a postmenopausal woman with two overlapping hereditary cancer predisposition syndromes: BRCA2‐associated hereditary breast and ovarian cancer syndrome and MLH1‐related Lynch syndrome, consistent with MINAS. MINAS refers to the coexistence of germline pathogenic variants in two or more cancer susceptibility genes in a single individual. Although rare, this entity is being increasingly recognized due to broader access to multigene panel testing and expanded use of exome/genome sequencing, particularly in populations with high genetic heterogeneity. Several studies have documented cases of MINAS involving BRCA1/2 and mismatch repair gene variants, highlighting variations in clinical presentation, tumor biology, and treatment responses. Whitworth et al. reported one of the earliest case series and systematic reviews of patients with multiple pathogenic germline variants in inherited cancer susceptibility genes, including a patient with concurrent BRCA1 and MLH1 variants and a higher frequency of synchronous and metachronous tumors. In their review, 82 cases involving 17 inherited cancer genes were identified, and BRCA1/BRCA2, BRCA2/TP53, BRCA/MLH1, and APC/MLH1 were the only combinations reported in more than one family [1]. This is relevant to our case because it supports the concept that dual germline pathogenic variants may underlie overlapping hereditary cancer phenotypes and multiple primary malignancies, as observed in our patient.

McGuigan et al. performed a systematic review including 385 individuals with 430 pathogenic or likely pathogenic variants across 63 cancer susceptibility genes. BRCA1/2 variants accounted for 78.5% of cases, and 28% of patients with MINAS had multiple primary tumors at presentation. The most frequent tumor combinations were breast–ovarian, breast–breast, and colon–colon [4]. This is relevant to our case because our patient also presented with multiple primary malignancies in the setting of a BRCA2/MMR‐associated MINAS phenotype, which is consistent with the increased burden of multiple tumors reported in this series.

Yuen et al. conducted a systematic review of 413 MINAS cases and found that carriers had younger cancer onset and higher rates of multiple malignancies than monoallelic and noncarriers. Combinations involving BRCA1/2 and Lynch syndrome genes were among the most frequent and were associated with both syndrome‐specific and atypical malignancies. Notably, a substantial proportion of patients developed multiple primary cancers or malignancies not fully explained by a single hereditary cancer syndrome [7]. This is relevant to our case because our patient harbored a BRCA2/MLH1 combination, one of the clinically important MINAS patterns highlighted in this review, and developed two distinct primary malignancies not fully explained by a single hereditary cancer syndrome.

A study of MINAS in the Mexican population by Aguilar et al. identified a higher prevalence (5.9%) than other cohorts (1%–3%). Among 2282 patients, 23 had MINAS, mostly linked to BRCA1, BRCA2, and MUTYH. Breast cancer, particularly triple‐negative, predominated (86.95%). The combinations of BRCA1/CHEK2, BRCA1/CDKN2A, and BRCA1/BRCA2 were the most frequent [8]. It is important in this case because breast cancer was the sentinel malignancy that prompted genetic evaluation in our patient, reinforcing that MINAS may first become clinically apparent through a BRCA‐associated breast cancer presentation.

Nevertheless, only a few case reports and studies exist describing patients with concurrent BRCA1/2 and mismatch repair protein alterations. Harada et al. reported a Japanese patient with ovarian mucinous adenocarcinoma who was found to carry germline pathogenic variants in MSH2 and BRCA2. Molecular analysis revealed microsatellite instability‐high status, and the tumor exhibited both BRCA2 loss of heterozygosity and mismatch repair deficiency, suggesting a dual mechanism of tumorigenesis [9]. This report is relevant to our case because it provides support for the coexistence of homologous recombination deficiency and mismatch repair deficiency within the same hereditary cancer context.

In 2022, Ozer et al. conducted a comprehensive study on young‐onset pancreatic and colorectal cancer patients with concurrent germline variants in BRCA1/2 and mismatch repair genes. Given the significant variability in tumor phenotypes and treatment responses, the study underscored the necessity of comprehensive germline and somatic characterization to inform therapeutic decisions. Patients in this cohort exhibited distinct tumor behaviors, ranging from aggressive pancreatic malignancies to more indolent colorectal cancers [10]. It supports the need for comprehensive germline and tumor characterization in patients with concurrent BRCA and mismatch repair gene variants, particularly when the clinical presentation spans more than one hereditary cancer syndrome, as in our case.

In conclusion, this case underscores the complexity of managing patients with MINAS, highlighting the critical need to integrate genetic insights into oncologic care. A multidisciplinary approach focusing on early detection, targeted therapies, and preventive strategies is essential for improving outcomes in this high‐risk population. Furthermore, the significant clinical challenges spanning oncology, genetics, surgery, and multidisciplinary care necessitate a nuanced understanding of overlapping syndromic manifestations, molecular pathways, and personalized therapeutic strategies. This case reinforces the importance of incorporating genomic profiling into treatment paradigms to optimize management strategies for patients with dual germline pathogenic variants.

## Funding

No funding was received for this manuscript.

## Consent

Patient consent was obtained.

## Conflicts of Interest

The authors declare no conflicts of interest.

## Data Availability

Data sharing is not applicable to this article as no datasets were generated or analyzed during the current study.
